# Metabolomics of Multimorbidity: Could It Be the *Quo Vadis*?

**DOI:** 10.3389/fmolb.2022.848971

**Published:** 2022-03-11

**Authors:** Pujarini Dash, Soumya R. Mohapatra, Sanghamitra Pati

**Affiliations:** ^1^ Regional Medical Research Centre, Bhubaneswar, India; ^2^ Department of Research and Development, Kalinga Institute of Medical Sciences, KIIT Deemed to Be University, Bhubaneswar, India; ^3^ School of Biotechnology, Kalinga Institute of Industrial Technology (KIIT), Deemed to Be University, Bhubaneswar, India

**Keywords:** metabolomics, multimorbidity, metabolic pathway, chronic diseases, therapeutic target

## Abstract

Multimorbidity, the simultaneous presence of two or more chronic diseases, affects the health care to a great extent. Its association with health care cost, more disability, and poor quality of life makes it a major public health risk. The matter of worry is that management of a multimorbid condition is complicated by the fact that multiple types of treatment may be required to treat different diseases at a time, and the interaction between some of the therapies can be detrimental. Understanding the causal factors of simultaneously occurring disease conditions and investigating the connected pathways involved in the whole process may resolve the complication. When different disease conditions present in an individual share common responsible factors, treatment strategies targeting at those common causes will certainly reduce the chance of development of multimorbidity occurring because of those factors. Metabolomics that can dig out the underlying metabolites/molecules of a medical condition is believed to be an effective technique for identification of biomarkers and intervention of effective treatment strategies for multiple diseases. We hypothesize that understanding the metabolic profile may shed light on targeting the common culprit for different/similar chronic diseases ultimately making the treatment strategy more effective with a combinatorial effect.

## Introduction

Multimorbidity, which is defined as the co-occurrence of two or more chronic conditions, has become a global health problem for health policy makers and health care providers. It often leads to functional limitations, visits to several medical specialists, and various therapies and treatments, potentially resulting in reduced quality of life. The true extent of multimorbidity is difficult to measure as there is no proper system for reporting, and the existing evidence base is also not complete. While data on most commonly occurring combination of diseases are scarce, it is known that multimorbidity is highly heterogenous, and patients can suffer from a wide range of different clusters of diseases. Similarly, very few information is available regarding modifiable factors that predict the risk of different types of multimorbidity. It is highly important to identify the presence of any environmental, behavioral, or biological factors that predict the risk of some types of multimorbidity independent of factors that modulate the risk of individual disease conditions. A study carried out by our group showed that among patients attending primary care settings in India, acid peptic disease with arthritis/chronic backache/tuberculosis/chronic lung disease were most leading diads among 18–29 age group, whereas the older population had more frequent combinations of hypertension + arthritis/chronic lung disease/vision difficulty and arthritis + chronic backache (Pati et al., 2017). Furthermore, the triad of acid peptic disease + arthritis + chronic backache was common in men in all age groups (Pati et al., 2017). Similarly, in a large-scale study conducted in the UK, Bisquera et al. reported the predominance of long-term conditions or diseases with other comorbidities, with the prevalence of these conditions alone, without any other comorbidity in a person was found to be on the lower end (Bisquera et al., 2021). Furthermore, during the ongoing COVID pandemic, categorization of patients according to their multimorbid conditions in treatment management of COVID-19 has become crucial. Of note, people with multimorbidities faced various challenges for their treatment and routine checkup because of pandemic-caused-restrictions, and on the other hand, multimorbidity was found to be prevalent among COVID-19 patients who lost their lives (Fernández-Niño et al., 2020; Pati et al., 2021a).

When different disease conditions present in an individual share common responsible factors, treatment strategies targeting at those common causes will certainly reduce the chance of development of multimorbidity occurring because of those factors. Multimorbidity is a major risk factor among psychiatric patients as half of them suffer from different multimorbid conditions and associated high health care utilization and expenditure (Pati et al., 2021b). However, the paucity of information about the most commonly occurring clusters of diseases and mainly those that do not seem to share any common etiology limits the possibility of developing therapeutic methods for treating related disease conditions at one go. Therefore, understanding the causal factors of simultaneously occurring disease conditions and investigating the connected pathways involved in the whole process are of high priority among public health researchers in the current time. Keeping sight of this necessity, investigations have focused on the “multi-omics” approaches to decipher the causal agents behind occurrence of multimorbid conditions, and here we delve specifically into metabolomics, one of the emerging “omics” arenas, to look for solutions to the emerging threat of multimorbidity.

## Association of Metabolites and Metabolomics With Multimorbidity

**FIGURE 1:**
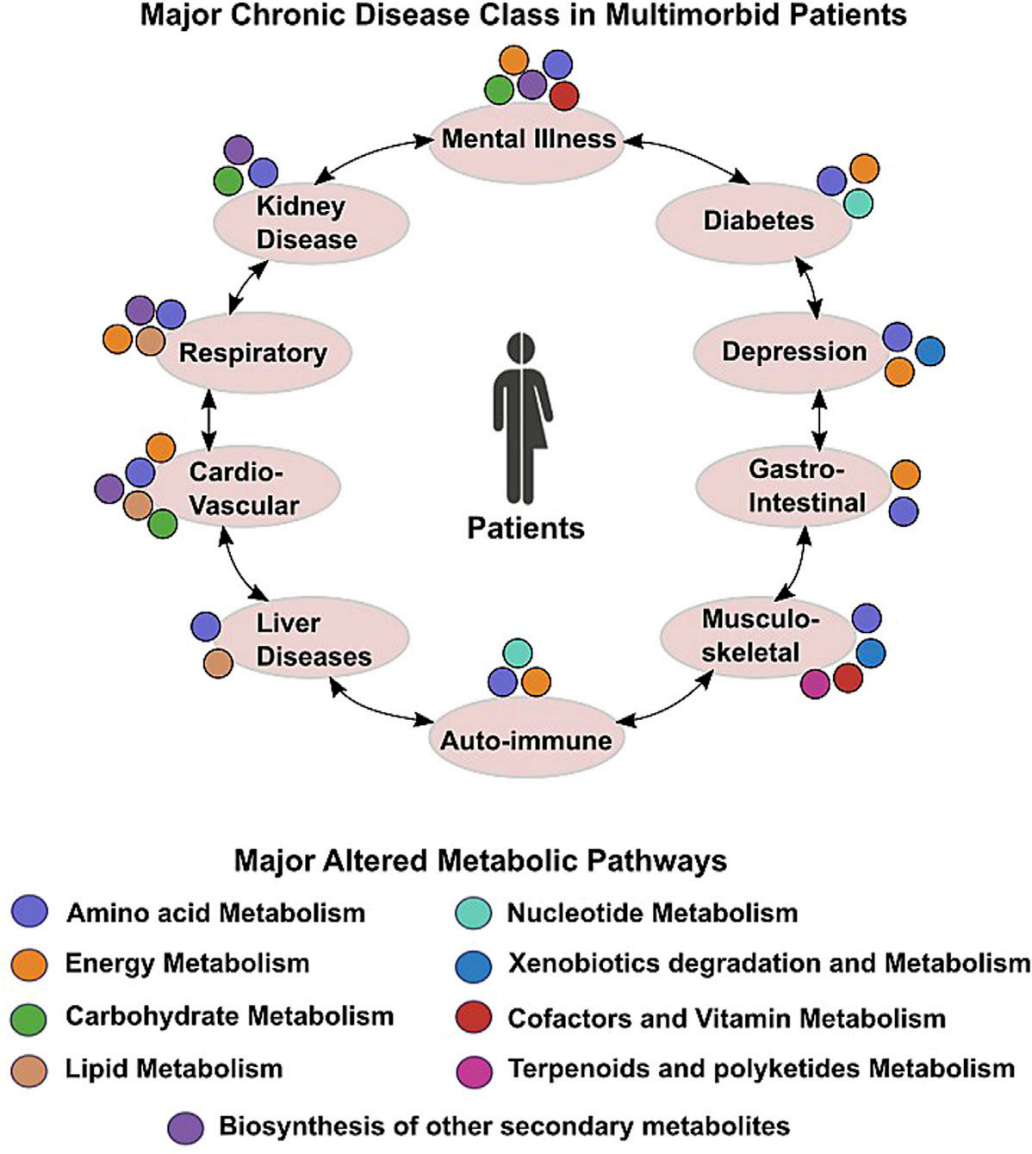
Scheme depicting altered metabolic commonalities in major chronic conditions found in multimorbid patients.

Hypothesis-free, untargeted investigations of in-depth molecular profiling and analysis of the association between metabolites and disease might address many unanswered questions related to unknown etiology and responsible factors for multiple chronic diseases. Understanding different metabolic pathways and their role in pathophysiology of different diseases have been most prolific pursuit in medical research. Metabolites play crucial role in growth, maintenance, and reproduction of organisms and serve as a great source of information for illustrating the underlying molecular mechanism of different diseases. The metabolome reflects changes in genome, transcriptome, and proteome showing real-time events happening inside living organisms. Metabolomic analysis or metabolomics is believed to be an effective technique for identification of biomarkers and intervention of effective treatment strategies for multiple diseases. There is plenty of evidence that diseases are associated with changes in the metabolic profile (Cheng et al., 2017), including long-term conditions such as autoimmune disorders such as multiple sclerosis, diabetes, and even cancers. The involvement of different metabolic pathways in different chronic diseases of multimorbid patients is presented in Figure 1. Therefore, it holds immense significance to study the metabolomics of diseases for early diagnosis and effective treatment.

A study in elderly adults found that the resting metabolic rate predicts future multimorbidity (Fabbri et al., 2015). A persistent increase in the metabolic rate is known to stimulate mitochondrial dysfunction and oxidative stress which in turn contributes organ damage and compromised health status, a reason sufficient enough for initiation of multiple chronic diseases (Fontana and Hu, 2014). Furthermore, evidences showing association of the PI3K/AKT pathway (the central pathway in the cellular metabolism) with atrial fibrillation, chronic obstructive pulmonary disease, heart failure, and multimorbidities suggest the plausible involvement of metabolic pathways in simultaneous presence of multiple chronic conditions (Fontana and Hu, 2014). According to Li et al., 2021, five comorbidities such as diabetes, obesity, chronic pulmonary obstructive disease (COPD), CKD, and obstructive sleep apnea share 299 common metabolites (Li et al., 2021). Apart from sharing common metabolic entities, a hallmark of most long-prevailing conditions is alterations in homeostatic metabolic pathways. Altered metabolic pathways in major ailments are easier to detect and remedy; however, subtle changes in homeostatic metabolic processes are a characteristic feature of chronic low-intensity ailments, thus avoiding scientific and medical scrutiny. However, how these metabolites play a role in occurrence of multimorbidities and whether understanding their metabolomics can pave a way for new, effective therapies, are yet to be fully comprehended.

## Can Metabolomics Solve the Question?

One of our previous works reveals that around 28% outdoor patients visiting primary health care centers in Odisha, India, have multimorbidities, and their diversified effects negatively affect health-related quality of life of patients (Pati et al., 2019). Management of a multimorbid condition is complicated by the fact that multiple types of treatment may be required to treat different diseases at a time, and the interaction between some of the therapies can be detrimental. This suggests that search for common mechanisms/molecules/pathways among the different disease conditions may be more useful as a common treatment procedure can be formulated to target commonly altered metabolic pathways. According to Burgel et al., 2010, the most common comorbidities of COPD patients, such as diabetes, chronic ischemic heart disease, metabolic syndrome, and metabolic bone disease, may share common molecular pathways (Burgel et al., 2010). It is possible that an existing treatment for a comorbidity may have significance in COPD treatment. For example, bioinformatics studies have shown that statins that reduce mortality in cardiovascular disease also seem to act in a similar way in COPD patients (Young and Hopkins, 2013). Metabolomics provides a great scope to understand the molecules/pathways involved/shared by different diseases and their association with those diseases. The co-occurrence of type-2 diabetes (T2D) and cardiovascular diseases is common, and its high degree of connectivity has been found with other diseases also (Guasch-Ferré et al., 2016). The lack of proper database and horizontal integration between health care providers providing treatment to patients with existing diseases make multimorbidity a random assortment of individual conditions. Now, the public health policy makers are making a point to consider multimorbidity as an accumulation of multiple predictable diseases in the same person.

In a study carried out by Pietzner et al. (2021), 420 metabolites were found to be associated with at least two different diseases or all-cause mortality, and 220 metabolites were specifically associated with one disease only (Pietzner et al., 2021). In the same study, a higher degree of interaction was observed among cardiometabolic and respiratory diseases including lung cancer, COPD, cerebral stroke, T2D, chronic heart disease, heart failure, and renal and liver diseases across different groups of metabolites (Lamming et al., 2013). Interestingly, the plasma level of N-acetylneuraminate was positively associated with 14 partly unrelated diseases, whereas many other metabolites including cotinine, N-acetylated amino acids, nucleotides, glycerophospholipids, products of microbial metabolism, catabolites of vitamin-C, heme degradation products, and sulfated steroids were strongly associated with multiple diseases (Lamming et al., 2013). The findings of common metabolites among related diseases as well as seemingly unrelated diseases indicate toward probable use of these metabolites as common treatment strategies or biomarkers in multimorbidity. The PI3K/AKT pathway is known as a master regulator of cellular metabolism, and its activation is directly correlated with the cellular metabolism. This pathway has been found to be associated with atrial fibrillation, chronic obstructive pulmonary disease, heart failure, and multimorbidities, and activated AMPK (central regulator of energy homeostasis) leads to extended life span and reduces diabetes, neurodegenerative diseases, and cardiovascular diseases in primates (Lamming et al., 2013; Li et al., 2021). These evidences suggest the plausible involvement of metabolic pathways in simultaneous presence of multiple chronic conditions, and their inhibition offers a future therapeutic opportunity.

Mitochondria are an important source of reactive oxygen species (ROS), and COPD is linked to increased ROS production by mitochondria. The expression of prohibitin 1 (PHB1), which is present in the mitochondrial membrane, is reduced in epithelial cells of smokers and COPD patients (Soulitzis et al., 2012). On the other hand, the PHB1 expression is reduced in endothelial cells, and its inhibition results in mitochondrial ROS production, cellular senescence, and impaired angiogenesis which is indicative of its link with cardiovascular diseases (Schleicher et al., 2008). Reported association of PHB1 with diabetes and insulin resistance also provides a mechanism which connects multimorbidity to mitochondrial dysfunction (Theiss and Sitaraman, 2011). Cells remove unnecessary products (degraded proteins, organelles, and foreign organisms, etc.) in order to maintain its normal function through a process called autophagy. Defective autophagy is also known to be involved in ageing, age-related diseases, and cardiovascular and metabolic diseases and might be the common pathway for multiple disease conditions and responsible for their clustering or multimorbidity in COPD patients (Schneider and Cuervo, 2014). Similarly, sirtuin is another important protein that plays key roles in cellular functioning. The expression of sirtuin 1 (SIRT1) is decreased in diseases of accelerated ageing including Alzheimer’s disease, chronic kidney disease, osteoporosis, T2D, metabolic syndrome, COPD, atherosclerosis, and cardiac failure (Nakamaru et al., 2009; Guarente and Franklin, 2011). It also induces autophagy by inhibiting mTOR (Lee et al., 2008). Looking at the involvement of this protein in multiple disease conditions, possibilities of using SIRT1 activators for age-related diseases cannot be overlooked. Recent reports on effective use of diabetes drugs such as empagliflozin and liraglutide to decrease cardiovascular risks in diabetic patients sheds light on the metabolic interconnection between diabetes, cardiovascular diseases, and obesity (Zinman et al., 2015; Marso et al., 2016). The effect of alteration in glucose, amino acid, fatty acid, and ion metabolism on different chronic diseases and how one condition can lead to another are explained schematically in Supplementary Figure S1.

**FIGURE 2:**
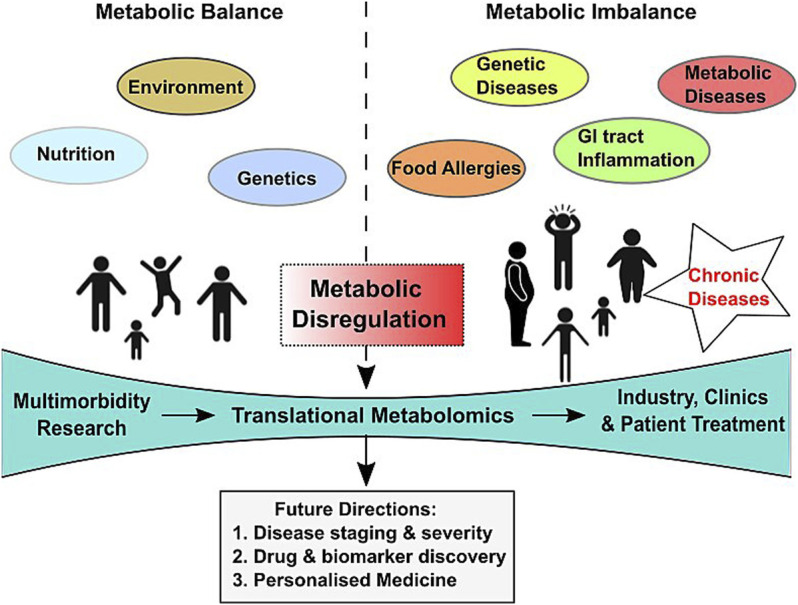
Scheme depicting influencing factors, model of care, and future directions of translational metabolomics in multimorbidity diagnosis and therapy.

Another usually slow-developing chronic condition is cancer, characterized by aberrant cell division by malignant cells. Persons with cancers are often comorbid with other chronic diseases, especially in patients from the lower socioeconomic status (Ogle et al., 2000). Chronic diseases that have often been found occurring as comorbid conditions in cancer patients are hypertension, hyperlipidemia, osteoarthritis, hypothyroidism, colitis, and diabetes (Roy et al., 2018). An aberrant metabolism is a well-known hallmark of malignant cancer cells, with cancer cells using altered metabolome to their advantage (Opitz et al., 2020). A majority of the previously comorbid conditions are known to alter homeostatic metabolic processes. In concurrence, these prevailing comorbidities in cancer patients often lead to significant reduction in overall survival by facilitating disease progression and hampering disease-free survival (Shieh et al., 2012)**.** However, knowledge about shared causal factors of unrelated diseases is scanty even in highly studied cancer entities. Metabolomics/molecular profiling can unravel the metabolite–disease association and the shared etiologies of different diseases that make up multimorbidity. The role of altered metabolites in multimorbidity and the future directions have been depicted in Figure 2.

## Role of the Gut Microbiota–Metabolites–Multimorbidity Axis in Therapy and Treatment

In a healthy human intestine, the microbes live as commensals, protect their host against pathogens, and regulate immune response and metabolism of carbohydrates and lipids. Commensal organisms produce different types of metabolites which may affect the host metabolic process with associated beneficial or pathogenic effects. The altered gut microbiome has been associated with an increasing number of diseases such as diabetes, complex gastrointestinal disorders (for e.g., inflammatory bowel disease), and colorectal cancer. Each of the bacterial community living inside the human gut can take part in metabolizing nutrients, bile acids, hormone secretion, and normal immune function of the host. The fecal metabolome may represent almost 68% of the variance in within an individual gut microbiome composition reflecting high degree of association between gut microbial structure and metabolic output of a person (Zierer et al., 2018). The relation between gut microbiome and obesity has already been established through a metabolite study. A metabolomic study on Chinese young obesity and lean people has found an obesity-related metabolite change in plasma with a high increase in glutamate in obese people. Of note, there was a relation between plasma glutamate and intestinal bacteria *Bacteroides thetaiotaomicron*, which had an association with obesity (Liu et al., 2017). On the other hand, obesity is a known risk factor for many other diseases including diabetes, several types of cancer, and cardiovascular disease (Guh et al., 2009). Trimethylamine-N-oxide (TMAO) is a metabolite produced by intestinal flora and known to promote cardiovascular disease (Wang et al., 2011). There is also another study confirming the role of intestinal flora-produced metabolites (choline, betaine, and TMAO) in cardiovascular diseases.

The intestinal flora is known to produce inflammatory signals and is responsible for creating inflammatory milieu and metabolic dysfunction (Sommer and Backhed, 2013). It is worth mentioning that microbial metabolites, short-chain fatty acids induce colonic inflammation, which is a major risk factor for inflammatory bowel disease and colon cancer (Zeng et al., 2019). Numerous evidences have linked gut microbiota to inflammatory diseases including Crohn’s disease, ulcerative colitis, multiple sclerosis, asthma, obesity, type-1 and type-2 diabetes, gastrointestinal cancers, and rheumatoid arthritis. Though association of gut microbiota with an individual disease condition has been studied to certain extent, its role in simultaneous occurrence of all those conditions/multimorbidity is yet to be unraveled. Metabolomic profiling of the patients may provide details of the molecules/products involved in the disease pathways and their link with gut microbiota.

## Discussion

The field of metabolomics is growing fast because of its application in identification of novel biomarkers for disease diagnosis, prognosis, treatment, and progression. Though it has given enormous insights and detail characterization of molecules and pathways involved in different diseases, lots of factors are there which act as hindrance in translating that information to clinics. Metabolomics of multimorbidity has great potential to develop new, effective, target therapies for treating related/seemingly unrelated diseases occurring simultaneously in an individual as it holds the strength to uncover the common metabolites/ pathways that connect different underlying pathogenic mechanisms. Additionally, more research on gut microbiota-derived metabolites and their role in multimorbidity may lead to a new treatment regimen involving administration of prebiotics, probiotics, and symbiotic supplementation can be derived as adjuvant therapy for CKD worldwide.

## Data Availability

The original contributions presented in the study are included in the article/Supplementary Material further inquiries can be directed to the corresponding author.
