# Supplementary figures and images for: Metabolomics of Multimorbidity: Could It Be the Quo Vadis?
Source: Front Mol Biosci. 2022 Mar 11;9:848971. doi: 10.3389/fmolb.2022.848971 (PMC8962190; doi:10.3389/fmolb.2022.848971)

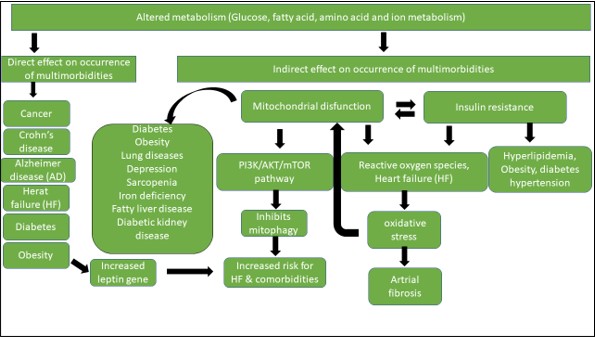

Supplement: Supplementary file 1 [file Image1.JPEG]
